# Estrogen Receptor 1 Inhibition of Wnt/β-Catenin Signaling Contributes to Sex Differences in Hepatocarcinogenesis

**DOI:** 10.3389/fonc.2021.777834

**Published:** 2021-11-22

**Authors:** Mamatha Bhat, Elisa Pasini, Chiara Pastrello, Marc Angeli, Cristina Baciu, Mark Abovsky, Angella Coffee, Oyedele Adeyi, Max Kotlyar, Igor Jurisica

**Affiliations:** ^1^ Ajmera Transplant Program, University Health Network, Toronto, ON, Canada; ^2^ Division of Gastroenterology & Hepatology, University of Toronto, Toronto, ON, Canada; ^3^ Institute of Medical Sciences, University of Toronto, Toronto, ON, Canada; ^4^ Toronto General Hospital Research Institute, University Health Network, Toronto, ON, Canada; ^5^ Osteoarthritis Research Program, Division of Orthopedic Surgery, Schroeder Arthritis Institute, University Health Network, Toronto, ON, Canada; ^6^ Krembil Research Institute, University Health Network, Toronto, ON, Canada; ^7^ Department of Pathology and University of Minnesota Medical Center, University of Minnesota, Minneapolis, MN, United States; ^8^ Department of Medical Biophysics, University of Toronto, Toronto, ON, Canada; ^9^ Department Computer Science, University of Toronto, Toronto, ON, Canada; ^10^ Institute of Neuroimmunology, Slovak Academy of Sciences, Bratislava, Slovakia

**Keywords:** estrogen, hepatocellular carcinoma, high-throughput, network analysis, Wnt/b-catenin, ESR1, interactome, PPIs

## Abstract

**Background:**

Hepatocellular Carcinoma (HCC) is a sexually dimorphic cancer, with female sex being independently protective against HCC incidence and progression. The aim of our study was to understand the mechanism of estrogen receptor signaling in driving sex differences in hepatocarcinogenesis.

**Methods:**

We integrated 1,268 HCC patient sample profiles from publicly available gene expression data to identify the most differentially expressed genes (DEGs). We mapped DEGs into a physical protein interaction network and performed network topology analysis to identify the most important proteins. Experimental validation was performed *in vitro* on HCC cell lines, in and *in vivo*, using HCC mouse model.

**Results:**

We showed that the most central protein, ESR1, is HCC prognostic, as increased ESR1 expression was protective for overall survival, with HR=0.45 (95%CI 0.32-0.64, p=4.4E-06), and was more pronounced in women. Transfection of HCC cell lines with ESR1 and exposure to estradiol affected expression of genes involved in the Wnt/β-catenin signaling pathway. ER-α (protein product of ESR1) agonist treatment in a mouse model of HCC resulted in significantly longer survival and decreased tumor burden (p<0.0001), with inhibition of Wnt/β-Catenin signaling. *In vitro* experiments confirmed colocalization of β-catenin with ER-α, leading to inhibition of β-catenin-mediated transcription of target genes c-Myc and Cyclin D1.

**Conclusion:**

Combined, the centrality of ESR1 and its inhibition of the Wnt/β-catenin signaling axis provide a biological rationale for protection against HCC incidence and progression in women.

## Introduction

Hepatocellular carcinoma (HCC) is a high-fatality cancer that develops in the context of chronic liver disease (1, 2). It is a cancer with sexual dimorphism that arises in a non-reproductive organ (3). In fact, men are up to 8 times more likely to develop HCC and have worse prognosis than women (2). Female sex is independently protective for HCC incidence [Hazard Ratio (HR) 0.75; 95%CI 0.65-0.86] and overall survival (HR 0.83; 95%CI 0.77-0.88) (4). A case-control study of 234 post-menopausal women treated for HCC showed that estrogen replacement therapy reduced incidence (HR 0.53; 95%CI 0.32-0.88) and was associated with better survival (HR 0.55; 95%CI 0.40-0.77) (5). Other retrospective studies suggest oral contraceptive use to be protective against HCC (6–8). These studies suggest that HCC biology is subject to sex hormone modulation (3, 4, 9–16).

ESR1 has been recognized as a tumor suppressor gene, with promoter hypermethylation being predictive of tumor progression (17). Its expression has been shown to inversely correlate with HCC tumor size and disease stage in a genome-wide expression analysis (18, 19), consistent with preclinical data demonstrating that loss of ESR1 accelerates carcinogenesis. Binding of estrogen to Estrogen Receptor-alpha and -beta (ER-α and -β) induces receptor dimerization and transcriptional regulation (20, 21). Hormone receptor dimers bind DNA at their specific response elements (22–25), or interact with chromatin-associated proteins to exert transcriptional activation of proliferation, cell cycle progression and cell survival (26). These data suggest an important role for ESR1 in incidence and progression of HCC, though it is unclear how ESR1 impacts cancer signaling pathways critical to HCC progression.

To further examine the role of estrogen receptor signaling in driving sex differences in hepatocarcinogenesis, we performed a comprehensive integrative analysis of publicly available HCC patient profiles and analyzed the resulting physical protein-protein interaction network. We discovered ESR1 to be a central gene in HCC pathogenesis, independently protective for survival in HCC in both men and women. Activation of ESR1 resulted in marked inhibition of Wnt/β-catenin signaling in both male and female mice with HCC. We finally interrogated the mechanistic basis of this effect *in vitro*, by demonstrating that colocalization of ER-α (protein product encoded by ESR1) with the transcription factor β-catenin along the Wnt pathway results in decreased transcription of target genes cyclin D1 and c-myc. The effect of ER-α in suppressing HCC tumor growth through inhibition of Wnt signaling, the most commonly dysregulated in HCC, provides a new contributory mechanism for decreased HCC incidence and progression in women.

## Materials and Methods

### Data Collection, Analysis, and Database Compiling

All available high-throughput gene expression microarray datasets related to HCC samples were downloaded from published datasets. All entries on PubMed since 2002, representing the advent of high-throughput profiling, until February 2017 were considered for inclusion. A second search was performed using Gene Expression Omnibus (GEO), a public functional genomics data repository containing high-throughput array data (https://www.ncbi.nlm.nih.gov/geo) covering all HCC high-throughput gene expression profiling datasets comparing HCC to adjacent non-tumoral tissue. These datasets publicly available on GEO and published in papers were analyzed using GEO2R (https://www.ncbi.nlm.nih.gov/geo/info/geo2r.html), a web tool available on the portal, to identify genes differentially expressed between samples of HCC *versus* the non-tumoral portion of the liver. GEO2R compares original submitter-supplied processed data tables using the GEOquery and limma R packages from the Bioconductor project (27–29). Following instructions available online at https://www.ncbi.nlm.nih.gov/geo/info/geo2r.html, all dysregulated genes were retrieved, and only those with an adjusted p-value p<0.05 and an expression fold-change value below ≤0.5 or above ≥1.5 were collected for further analysis. We also included genes from 17 papers presenting and validating gene signatures (**Supplementary Figure 1** and **Supplementary Table 1**).

The study workflow is illustrated and reasons for study exclusion are detailed in **Supplementary Figure 1**. The included papers (n=36) compared gene expression profiling in human HCC tissue *versus* the non-tumoral liver tissue in the same patient. These were obtained from 19 papers with datasets and 17 gene signature papers. This comprised data across 1,268 HCC patient samples and 1,402 controls (**Supplementary Figure 1**). Gene expression profiling had been performed using Affymetrix and Illumina microarray platforms, as detailed in **Supplementary Table 1**. The list of dysregulated genes for 19 datasets was obtained using GEO2R (p-value<0.05), and an integrative network analysis was performed on this data as described below. The genes from the 17 signatures and their modulations listed in the papers were collected and curated to serve as independent validation of the HCC relevance for the genes identified in the network analysis.

Available patient data, including sex, etiology of liver disease (Hepatitis C, Hepatitis B, alcohol, fatty liver disease) on the basis of which the HCC tumors developed, presence of cirrhosis, the Model for End-stage Liver Disease score (MELD score, an assessment of the severity of liver dysfunction), tumor histology, stage of cancer, alpha-fetoprotein (AFP) level, overall and recurrence-free survival following treatment were also documented.

### Network Construction

A Protein interaction network was constructed using NAViGaTOR 3.0.13 (http://ophid.utoronto.ca/navigator), a visualization application, wherein networks are represented as annotated graphs and defined by nodes (for example, genes or proteins) and edges (the relationships between the proteins, such as physical protein interactions)^12^. In our study, nodes are proteins, annotated with Gene Ontology Molecular Function (node color), and edges represent direct physical interactions from IID database (http://ophid.utoronto.ca/iid) (30).

### Calculation of Network Centrality

For all differentially expressed genes reported with a consistent modulation in at least 10 out of 19 publications, we retrieved the known interactors using the Integrated Interactions Database (http://ophid.utoronto.ca/iid) (30) version 2017-04, selecting only human experimentally-detected, physical protein-protein interactions, whose encoding genes are expressed in liver tissue. Interactors reported several times were highlighted.

Betweenness centrality was calculated in a protein-protein interaction network comprising the most frequently dysregulated genes (proteins) and their experimentally detected interaction partners annotated with human liver using the betweenness function in the igraph library version 1.0.1, in R version 3.3.1 (31). This network was visualized and analyzed in NAViGaTOR (32) ver. 3.0.13.

### HCC Mouse Model

A genetic mouse model of HCC was generated through hydrodynamic tail vein injection of proto-oncogene-coding plasmids (33). Twenty µg of pT3-EF5a-G12D-mutant-K-Ras and pT3-EF5a-S45Ymutant-β-catenin-Myc-Tag along with Sleeping Beauty transposase (a kind gift from Dr. X. Chen at University of California, San Francisco) in a ratio of 25:1 was diluted in 2 ml of 0.9% NaCl, filtered through a 0.22 µm filter, and injected into the lateral tail vein of 8-10-week-old FVB/N mice [Charles River (Wilmington, MA)] in 5-7 seconds (33, 34). The mice were housed in the animal facility at the University of Toronto. The experimental protocol was approved by the Institutional Animal Care and Use Committee at the University of Toronto. Mice were fed a standard diet and monitored according to the animal committee’s regulations Prior to establishment of HCC tumors, animals were started at 2 weeks post-injection on a 5-week treatment as follows: 1) ER-a agonist Propyl pyrazole triol (PTT) subcutaneously at 1mg/kg (Sigma-Aldrich H6036) N=6; 2) ER-a antagonist methyl-piperidino-pyrazole (MPP) given subcutaneously at 200ug/kg (Sigma-Aldrich M7068) N=6; and 3) Control N=4. All animals were carefully monitored for signs of morbidity or discomfort. Animals were euthanized at 10 weeks after injection or at the earliest signs of morbidity. Body weights and liver weights were recorded. Gross images were obtained at the time of sacrifice to document macroscopic tumors. The liver was collected for histology and RNA extraction purposes. Microscopic tumor burden was assessed on H&E-stained sections. All the samples were cut across and the surface of each section occupied by tumor was estimated at low magnification (2.5X) by the pathologist who was blinded to the groups. Kaplan-Meier survival analysis was performed with GraphPad Prism ver. 8 (GraphPad Software, La Jolla, California).

### Mouse Gene Expression Array

RNA was obtained from N=2 animals per group, (agonist and control both male and female) from each group using RNeasy mini kit from Qiagen.

Integrity of DNA and RNA was assessed by Agilent 2100 Bioanalyzer (Agilent, Santa Clara, CA, USA). 500 nanograms of RNA were used for analysis with Affymetrix Mouse Gene 2.0 ST platform (Thermo Fisher, Waltham, MA). Raw array data were processed and analyzed using the Affy package in R included in the Bioconductor package (version 3.6).

Transcript profiling: Gene expression data is available at Gene Expression Omnibus (GEO), (https://www.ncbi.nlm.nih.gov/geo) with accession GSE167175.

### Validation in Patient Samples

To determine whether the central gene ESR1 had prognostic value, we used KMplotter on liver cancer RNA-seq data (n=364 patients), a web-based tool that enables survival analysis across multiple cancers and datasets (35). KMplotter compares two patient cohorts by plotting Kaplan-Meier curves and calculating a hazard ratio and log-rank P value. Patient samples were split into two groups according to auto selection of the best cutoff for ESR1 (RNAseq probe ID 2099), in order to assess its prognostic value. We ran multivariate overall survival analysis based on the high *versus* low expression of ESR1 in tumors. The two groups were compared by a Kaplan-Meier survival plot, and the hazard ratio with 95% confidence intervals and log-rank p-value were calculated.

### Statistical Analysis

Data was presented as mean with standard deviation. GraphPad Prism software ver. 8 was used for all statistical calculations (GraphPad Software, La Jolla, California). Two-group comparisons were performed using unpaired two-tailed Student’s t-tests, for more than two groups comparison we used one-way ANOVA followed by Tukey’s *post-hoc* test. Survival curves analysis was performed using Gehan-Breslow-Wilcoxon test.

*Ethics Approval*: Animal studies were conducted under the approval of local Animal Care Committees (University of Toronto AUP 20012289, University Health Network AUP 6105), following Canadian Council on Animal Care (CCAC) guidelines.

Additional materials and methods used in this study are explained in the **Supplementary Material.**

## Results

### Literature Search Results

We identified 5,406 abstracts retrieved by searching PubMed for high-throughput gene expression data on human HCC samples, published between January 2002 and December 2017. The flowchart outlining the selection process is detailed in **Supplementary Figure 1**. Details regarding the included studies are provided in **Supplementary Table 1** and made available as a database of curated cancer gene expression signatures, annotated with their demographic and clinical characteristics, at the liver page of the Cancer Data Integration Portal (CDIP) (http://ophid.utoronto.ca/CDIPLiver).

### Integrative Analysis Reveals ESR1 as a Central Player in HCC Pathogenesis

**Figure 1:**
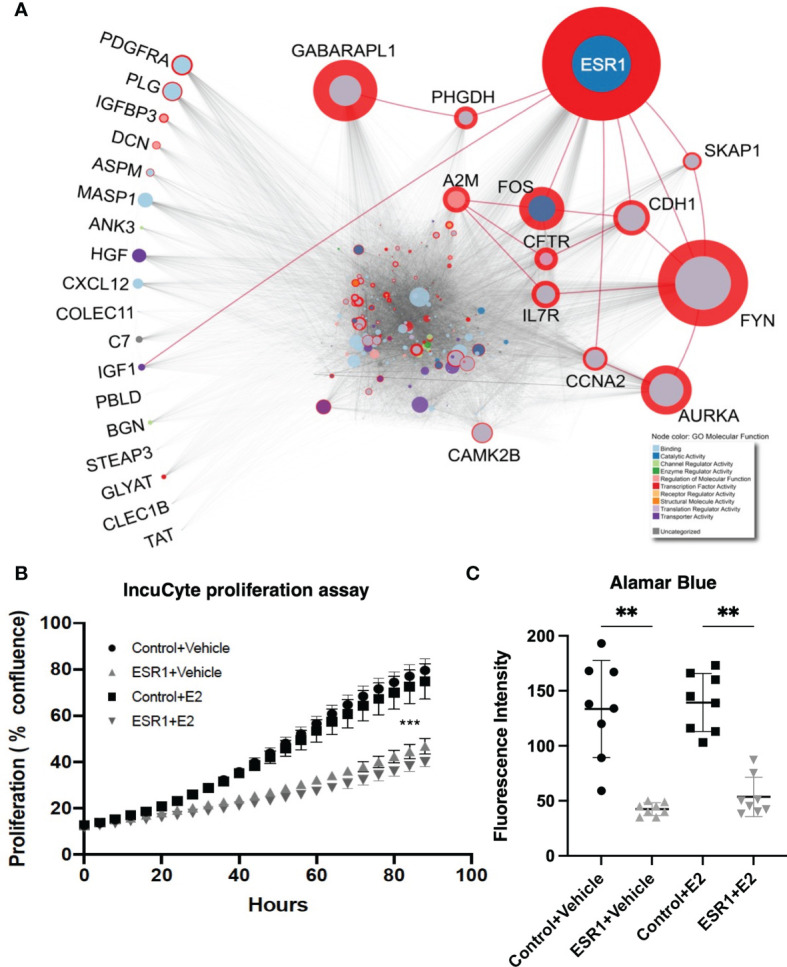
ESR1 is the most central node in the HCC protein-protein interaction network, and its overexpression affects HepG2 cells proliferation and viability reflecting its importance in HCC pathogenesis. **(A)** HCC protein-protein interaction network. The diameter of the red circle is proportional to the centrality of the protein. The size of the circles is proportional to the degree of the protein. Gene Ontology molecular function of the proteins is highlighted as node color, shown in the legend in the bottom-right corner. Red edges highlight direct interaction of ESR1 with other most central proteins. The proteins listed on the left are those dysregulated most commonly in the 19 datasets and with consistent modulation. Despite being the most commonly dysregulated, none of these are central to the HCC PPI network. **(B)** Results from the IncuCyte proliferation assay. ESR1-overexpressing cells proliferated significantly less than the control cells. Images were acquired every four hours for up to 90 hours. Mean with SD values from N=3 independent experiments are represented in the graph. ***p < 0.001, One-way ANOVA, Tukey correction for multiple comparisons. **(C)** Viability assay with Alamar Blue performed in n=3 independent experiments at 72 hours after transfection shows significant decrease of cell viability in cells transfected with ESR1 compared to the control vector Mean with SD values from N=3 independent experiments are represented in the graph. **p < 0.01, One-way ANOVA, Tukey correction for multiple comparisons. SD, standard deviation.

Integrative network analysis of high-throughput gene expression data revealed genes most frequently altered in HCC, along with the direction of change (**Supplementary Table 2**). We identified the protein-protein interactions of the top genes (considering deregulation in greater than half the number of publications) using IID (36, 37), and prioritized top-genes with at least 250 interactions (n=27), due to the exponential increase in number of proteins with a degree lower than 250 (**Supplementary Figure 2**). Betweenness centrality was calculated using a liver-specific network comprising the 27 proteins and their experimentally-detected interaction partners (**Figure 1A** and **Supplementary Table 3**), thereby identifying the most important proteins in the HCC network. ESR1 had the highest betweenness centrality, suggesting its importance in the HCC pathology, and indicating a potentially important role in hepatocarcinogenesis.

### ESR1 Overexpression Affects Proliferation and Viability of HCC Cells

To evaluate the impact of ESR1 overexpression, the HepG2 cell line was transfected with the ESR1 plasmid. Transfected cells were then exposed to estradiol (E2) or vehicle and monitored for 90 hours using the IncuCyte Zoom™ live cell imaging platform. Proliferation was monitored by analyzing the area occupied by cells (percentage confluence) transfected with ESR1 or pCMV control vector and exposed to E2 or vehicle. The confluence of cells growing in normal conditions for up to 90 hours showed that ESR1 overexpression significantly reduces cell proliferation (p<0.0001, one-way ANOVA followed by Tukey’s *post-hoc* test). The antiproliferative effects of ESR1 were strongly evident in HepG2 cells, and the addition of the ligand further improved the antiproliferative effect (**Figure 1B**).

The decrease in fluorescence detected by the Alamar blue between cells overexpressing ESR1 and receiving the control vector was significant (p=0.0019, one-way ANOVA followed by Tukey’s *post-hoc* test), even without the addition of the natural ligand E2 (p=0.0016, one-way ANOVA followed by Tukey’s *post-hoc* test), confirming that ESR1 expression directly affects cell viability (**Figure 1C**).

### Gene Expression Array

Upregulation of ESR1 resulted in significant up- or downregulated 365 genes (abs(FC)>1.5) as compared to control, of which 175 were protein-coding (**Supplementary Table 4**). We submitted these genes to pathDIP, in order to identify significantly enriched pathways (http://ophid.utoronto.ca/pathDIP) (38). 133 of the genes were annotated with at least one pathway. 83 pathways were significantly enriched with False Discovery Rate (FDR) < 0.05. The cancer-associated list of pathways is provided in **Table 1**. ESR1 upregulation significantly affected the Wnt/β-catenin signaling pathway (FDR=2.05E-06). The complete list is provided in **Supplementary Table 5**.

**Table 1:** ESR1 overexpression modulated genes associated with cancer pathways.

Cancer-related pathways	q-value (FDR: BH-method)
Formation of β-catenin: TCF transactivating complex	2.35E-08
Senescence-Associated Secretory Phenotype (SASP)	3.65E-07
RHO GTPases activate PKNs	3.21E-08
Oxidative Stress Induced Senescence	5.82E-06
Cellular Senescence	6.94E-07
TCF dependent signaling in response to Wnt	9.09E-08
Signaling by Wnt	2.05E-06
Tumor Necrosis Factor Pathway	2.46E-03
Viral carcinogenesis	8.64E-05

FDR, False discovery Rate; BH, Benjamini-Hochberg.

### ER-α Agonist Exposure Increases Survival in HCC Mice

**Figure 2:**
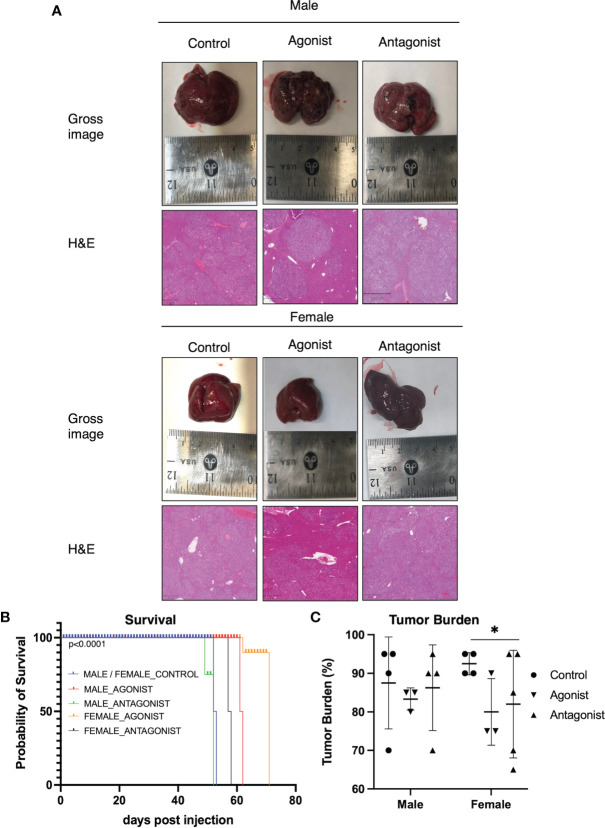
ER-α agonist exposure improves survival and decreases tumor burden **(A)** Gross images of livers from mice upon sacrifice show notable macroscopic disease in all groups. H&E staining of representative liver sections from the three groups in both male and female mice. Control group livers show more malignant nodules infiltrating the tissue. The agonist groups have smaller foci of tumor nodules (Magnification: 100X). **(B)** Kaplan-Meier curve comparing survival of HCC control mice (N = 4) and mice injected with ER-α agonist (N=6) or antagonist (N = 6). Agonist mice show significantly increased survival as compared to the control groups. p < 0.0001, Gehan-Breslow-Wilcoxon test. **(C)** Bar plot depicting tumor burden expressed as percentage of tumor cells in the liver tissue. Control groups livers show a higher percentage of infiltrating malignant cells. Mean with SD values are represented in the graph. *P < 0.05, multiple t-test. SD, standard deviation.

To investigate the effect of ER-α activation or inhibition in HCC, we used a well-described HCC mouse model (33). The control and antagonist mouse livers, in both male and female groups, had multiple HCC nodules infiltrating the liver as reported in **Figure 2A**. In contrast, the female agonist group showed smaller isolated tumor foci. ER-α activation through agonist treatment significantly improved survival (**Figure 2B**) by an additional 10 days for male and up to 14 days for female mice respectively (p<0.0001, Gehan-Breslow-Wilcoxon test). Mice exposed to ER-α antagonist showed decreased survival in male mice (48 days for antagonist mice compared to 52 days for the control group). The same treatment in the female mice did not shorten their lifespan (University Health Network Animal Use Protocol 6105). Control mice showed a tumor burden involving 85% of their livers, while the male mice treated with ER-α agonist showed tumor burden decreased to 78%. The decrease in tumor burden was even more pronounced (equal to 67%) in the female group mice treated with ER-a agonist (**Figure 2C**, p<0.05 multiple t-test).

### ER-α Agonist Treatment Inhibited Wnt/β-Catenin Signaling

Given the differences in survival and tumor burden with ER-a agonist treatment, gene expression profiling was performed to compare the agonist *versus* control mice. A higher number of dysregulated genes were identified in the female mice treated with ER-α agonist, with 266 upregulated and 280 downregulated genes (log_2_FC>1 or log_2_FC<-1, p value<0.05), while male mice treated with ER-α agonist had 173 upregulated and 64 downregulated genes compared to male control group. Pathway enrichment analysis was performed separately for the up- and down-modulated genes (**Table 2** and **Supplementary Tables 6, 7**). Female mice treated with ER-α agonist had marked enrichment in the Wnt/β-catenin signaling pathway, followed by mTOR and Hippo signaling pathways (**Supplementary Tables 6, 7**). The male mice showed a similarly marked enrichment for downmodulated genes involving the Wnt signaling pathway, followed by MAPK.

**Table 2:** ESR1 agonist affects genes involved in crucial HCC pathways.

Agonist *vs* Control (Female) Upregulated genes (n=266)	
Pathway Name	q-value (FDR: BH-method)
Nucleosome assembly (linked to histone)	3.27E-24
Mismatch repair	5.25E-11
Regulation of TP53 Activity through Methylation	3.57E-02
PRC2 methylates histones and DNA	1.19E-06
**Agonist *vs* Control (Female) Downregulated genes (n=280)**	
Wnt-β-catenin	1.53E-07
DNA replication initiation	2.09E-04
Hippo signaling	1.58E-02
PI3K_AKT_MTOR	3.44E-02
Wnt/Beta-catenin	1.53E-07
DNA replication initiation	2.09E-04
Hippo signaling	1.58E-02
**Agonist *vs* Control (Male) Upregulated genes (n=173)**	
Interleukin-3, Interleukin-5 and GM-CSF signaling	2.53E-02
**Agonist *vs* Control (Male) Downregulated genes (n=64)**	
JNK MAPK Pathway	4.25E-02
Wnt Signaling	2.53E-02

ESR1 agonist upregulates genes involved in Methylation and DNA mismatch repair and downregulates genes involved in cancer progression pathways, such as Wnt-β-catenin signaling, PI3K-mTOR, DNA replication and Hippo pathway. FDR, False discovery Rate; BH, Benjamini-Hochberg.

### ER-α Agonist Exposure Decreases Tumor Proliferation

To evaluate if the downmodulation in Wnt/β-catenin signaling identified by gene expression affected HCC cell proliferation, mice treated with ER-α agonist were evaluated for β-catenin, cyclin-D1 and Ki67 IHC staining as a reflection of tumor proliferation rate in comparison to controls. The overall decrease of β-catenin in the agonist mice compared to control (**Supplementary Figure 3A**), was followed by a decrease in the intensity of its target proteins cyclin-D1 and Ki67, both known markers of DNA replication and cell proliferation (**Supplementary Figures 3B, C**).

Further, we found that mice treated with ER-α agonist showed an overall different cellular positivity and distribution of ER-α compared to the control mice, supporting the hypothesis that activation of the receptor was responsible for its nuclear translocation (**Supplementary Figure 3D**). Moreover, there was increased nuclear positivity for both β-catenin (**Supplementary Figure 4A**) and ER-α (**Supplementary Figure 4B**) with concomitantly decreased nuclear positivity of cyclin-D1 (**Supplementary Figure 4C**) in the mice treated with ER-α agonist compared to control.

### ER-a Agonist Downmodulated ESR1 Interactor Genes Involved in Wnt/β-Catenin

To better understand the molecular connection between Wnt-β-catenin and ER-a, the protein interactors of ER-α were retrieved. 66 protein members of Wnt signaling are interactors of ER-a. Among these interacting proteins was β-catenin (*CNNTB1*), a crucial member of the nuclear transcription machinery (**Supplementary Figure 5A**). Moreover, *CCND1* and *MYC* were identified as target genes of β-catenin (**Supplementary Table 8**). HepG2 cells overexpressing ESR1 (FC=4.85, p<0.0001 Student’s t-test) (**Supplementary Figure 5B**) showed a decreased expression in β-catenin-transcribed genes like *CCND1* (FC=0.31 p=0.0019 Student’s t-test) and *MYC* (p=0.0023, Student’s t-test) (**Supplementary Figures 5C, D**).

### ER-α Colocalization With β-Catenin Decreases Target Gene Expression

**Figure 3:**
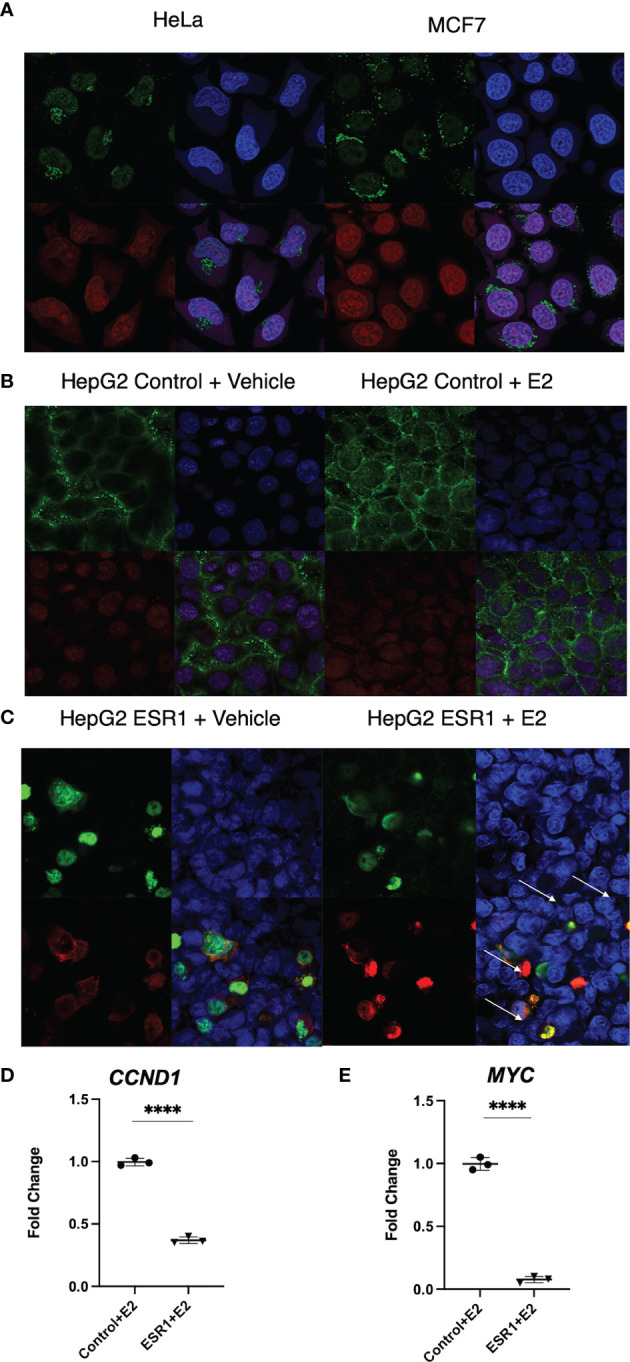
ER-α colocalization with β-catenin decreases β-catenin target gene expression *in vitro.* **(A)** Immunofluorescent analysis of β-catenin (top left panel) and ER-a (bottom left panel) on HeLa and MCF7 cells. Nuclei (top right panel) were stained with DAPI. Merged image (bottom right panel) showing a nuclear localization of ER-a in MCF7 and cytoplasmic localization of β-catenin in both cell lines. The images were captured at 40X magnification. **(B)** Immunofluorescent analysis of β-catenin (top left panel) and ER-α (bottom left panel) on HepG2 cells transfected with the control vector and exposed to vehicle or E2. Nuclei (top right panel) were stained with DAPI. Merged image (bottom right panel) showing the detection of cytoplasmic localization of β-catenin and absence of ER-α in the nucleus of HepG2 cells. **(C)** Immunofluorescent analysis of β-catenin (top left panel) and ER-α (bottom left panel) on HepG2 cells transfected with ESR1 coding vector and exposed to vehicle or E2. Nuclei (top right panel) were stained with DAPI. Merged image (bottom right panel) showing the concomitant detection in the nucleus of both β-catenin and ER-α in HepG2 cells represented by the yellow fluorescence as indicated by the white arrows. The images were captured at 40X magnification. **(D)** HepG2 cells overexpressing ESR1 expressed less *CCND1* (FC = 0.3), MYC (FC = 0.065). Mean with SD values from N = 3 independent experiments are represented in the graph. ****p < 0.0001, Student’s t-test. SD, standard deviation.

Cytoplasmic localization of β-catenin and ER-α in HeLa and MCF7 cells by confocal microscopy was used as positive control (top left panel **Figure 3A**). ER-a was detected in the nucleus of MCF7 (bottom left panel **Figure 3A**). HepG2 transfected with the empty vector showed cytoplasmic localization of β-catenin with no concomitant ER-α (**Figure 3B**). HepG2 transfected with ESR1 vector and exposed 72 hours to E2 had evidence of colocalization of ER-α and β-catenin (yellow signal as indicated by the white arrows in the bottom right panel, **Figure 3C**). There was significantly decreased expression of the β-catenin-transcribed genes *CCND1* (p<0.0001, Student’s t-test) and *MYC* (p<0.0001, Student’s t-test) by real-time PCR.

### ESR1 Overexpression Reduces β-Catenin Transcriptional Activity

**Figure 4:**
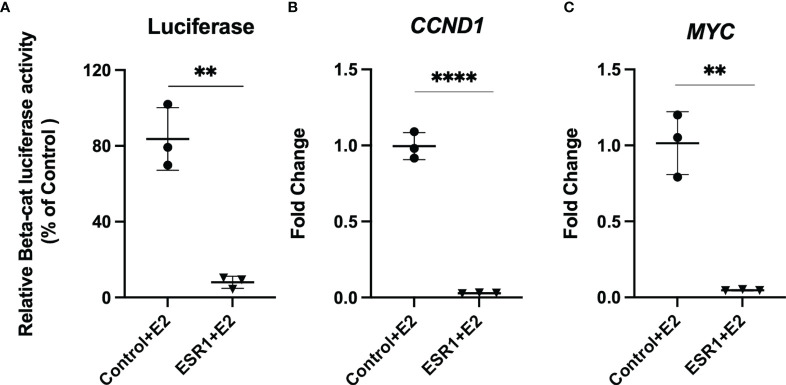
ESR1 overexpression decreases transcriptional activity and gene expression of β-catenin and Wnt/β-catenin signaling targets. **(A)** Representative bar graphs showing reduction of β-catenin transcriptional activity, as measured by luciferase assay in HepG2 cells following ESR1 co-transfection or control vector and exposure to E2 or vehicle. **(B)** Representative bar graphs showing reduction of mRNA expression of *CCND1* in HepG2 cells as measured by Real-Time PCR. **(C)** Representative bar graphs showing reduction of mRNA expression of MYC in HepG2 cells as measured by Real-Time PCR. Mean with SD values from N = 3 independent experiments are represented in the graph. **p < 0.01, ****p < 0.0001 Student’s t-test. SD, standard deviation.

Given that colocalization experiments revealed ESR1 translocation into the nucleus and colocalization with β-catenin, we evaluated whether this alters transcription by β-catenin of the target genes *CCND1* and *MYC*. Thus, we evaluated if overexpression of ESR1 and exposure to E2 could downregulate β-catenin transcriptional activity compared to HepG2 cells transfected with control vector (p=0.0015). (**Figure 4A**). Using Real-Time PCR analysis, we determined that ESR1 overexpression in the presence of E2 effectively downregulated the mRNA expression of β-catenin target genes such as *CCND1* (FC=0.02, p< 0.001, Student’s t-test) (**Figure 4B**) and *MYC* (FC=0.05, p=0.0013, Student’s t-test) (**Figure 4C**).

### ESR1 Is Prognostic of HCC Survival, Independent of Sex and Race

**Figure 5:**
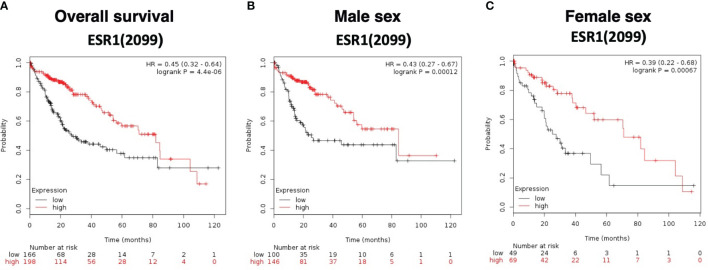
Survival curves based on expression of ESR1 (RNAseq ID 2099) in the TCGA dataset. **(A)** Overall survival according to ESR1 status based on auto selected best cutoff value (79). **(B)** Survival according to ESR1 status and further stratified by Male sex, based on auto selected best cutoff value (79) **(C)** Survival according to ESR1 status and further stratified by Female sex based on auto selected best cutoff value (43). Separation of patients by ESR1 expression in Kaplan-Meier plotter was based on the best cutoff computed from all the possible cutoff values between the lower and upper quartile (39).

Overall survival analysis was evaluated using The Cancer Genome Atlas (TCGA) HCC RNAseq data normalized with DESeq. ESR1 was significantly predictive of overall survival in 364 HCC patients with a significantly decreased hazard ratio of 0.45 (95%CI 0.32-0.64, log-rank p=4.4E-06), as shown in **Figure 5A**. Patients with high ESR1-expressing tumors had a median survival of 81.9 months, as compared to the cohort with low ESR1-expressing tumors at 27.6 months. This finding was independent of sex: overall survival was significantly improved in both males (HR 0.43 (95%CI 0.27-0.67, log-rank p=0.0001) (**Figure 5B**) and females (HR 0.39 (95%CI 0.22-0.68, log-rank p=0.0007) when ESR1 was upregulated (**Figure 5C**). Furthermore, we identified ESR1 to be significantly predictive of recurrence-free survival (HR 0.54 (95%CI 0.43-0.79, log-rank p=0.00044)) and progression-free survival (HR 0.4 (95%CI 0.25-0.63, log-rank p=3.6E-05)) (**Supplementary Figure 6**). Separation of patients by ESR1 expression in Kaplan-Meier plotter was based on the best cutoff computed from all the possible cutoff values between the lower and upper quartile (39).

## Discussion

It has been well established that women are protected against HCC incidence and progression in comparison to men, independent of environmental exposures (40). This significant impact of sex on HCC incidence and survival has been ascribed to hormones, though the mechanistic basis has not been well delineated. In this study, we substantiate these sex-specific differences in HCC by demonstrating that ESR1, and therefore, the estrogen-mediated effect is an important contributor to sex differences in HCC. We employed the betweenness centrality measure on the HCC protein-protein interaction network obtained through a comprehensive integrative analysis of all publicly available gene expression data in HCC. *In vivo* validation confirmed that ER-α agonism decreases HCC tumor burden and enhances overall survival. This was accompanied by significant inhibition of the Wnt/β-catenin signaling cascade, a critical pathway in hepatocarcinogenesis. Additionally, we demonstrated through knockdown *in vitro* experiments that the mechanistic basis of ESR1’s effect is through its colocalization with β-catenin, thereby preventing transcription of the pro-oncogenic genes c-myc and cyclin D1. Thus, using a network analysis approach, we demonstrate ESR1 as being important to HCC pathogenesis through inhibition of Wnt signaling, and more specifically through inhibition of β-catenin transcriptional activity, thereby providing a molecular explanation for women being protected against HCC incidence and progression.

These findings are in line with previous literature on female sex being independently protective for HCC incidence in patients aged 18 to 44 years (HR 0.75; 95%CI 0.65-0.86, p<0.001) (17). Overall survival in women with HCC was also significantly higher, with a hazard ratio of 0.83 (95%CI 0.77-0.88) (17). This male predominance of HCC has led to questioning the importance of sex hormones in HCC pathogenesis. A case-control study of 234 female patients treated for HCC showed that estrogen replacement therapy reduced risk (HR 0.53; 95%CI 0.32-0.88) and decreased the risk of death from HCC (HR 0.55; 95%CI 0.40-0.77, p=0.01) (41). Median survival was also significantly higher in estrogen users (33.5 months) as compared to non-users (24.1 months, p=0.008) (5). A retrospective study of over 3,000 patients determined that oral contraceptive use was associated with increased survival in women with HCC (5).

Susceptibility to HCC has also been demonstrated with specific ESR1 polymorphisms, as evidenced by a study of 248 patients with HCC compared to controls. There was a greater than two-fold increase in susceptibility to HCC when one of three ESR1 polymorphisms were found (42). ESR1 has been identified as a tumor suppressor protein whose expression inversely correlates with tumor size and disease stage, based on genome-wide expression and microRNA analyses (18). An epigenetic study of HCC also confirmed ESR1 as a tumor suppressor gene, with up to 83% of HCC patient tumors demonstrating ESR1 promoter hypermethylation (5) and predicting tumor progression (17). Variant ESR1 expression is predominant in male patients with hepatitis B-associated HCC and is predictive of worse survival (18, 19, 41).

ESR1 is a ligand-dependent transcription factor that, upon binding to estrogen, recruits coregulatory proteins and binds regulatory DNA sites containing an estrogen responsive element. This alters transcription of genes relevant to tumorigenesis and the immune response (43, 44). *In vitro* and *in vivo* data have demonstrated that estrogen inhibits HCC growth through upregulation of the JAK-STAT pathway (16), suppression of Interleukin-6 secretion, decreased hepatocyte growth factor production (13, 45), suppression of PPARa-associated hepatocarcinogenesis (46, 47), and inhibition of NF-KB (48). Specific effects of estrogen on the immune cells within the HCC tumor microenvironment have also been shown *in vivo (* 49, 50). Estrogen repressed HCC growth through inhibition of tumor-associated macrophages and the NLRP3 inflammasome (49). Conversely, in a preclinical model of diethylnitrosamine-induced HCC, loss of ESR1 was shown to accelerate hepatocarcinogenesis (51).

*Our in vivo* validation revealed that ER-α particularly inhibits the Wnt signaling cascade. In terms of processes, ESR1 significantly impacted transcription regulation, histone methylation, DNA repair, cell cycle and senescence. The effect of ESR1 on these processes is compatible with the literature on the impact of ESR1 in other cancers (52–60), with impact on transcriptional regulation (61), cell cycle, and tumor suppression (62). Interestingly, ER-α antagonist did not produce the opposite effect, but rather resulted in tumor burden similar to the control conditions. This again suggests that the positive effects of ER-α agonism are through inhibition of β-catenin transcriptional activity, without any graded effect once ER-α is inactivated.

Although Wnt signaling is the dominant pathway dysregulated in 40% of HCC tumors (15, 63), the current first-line therapies of lenvatinib or atezolizumab and bevacizumab target other less crucial pathways such as Ras/Raf/MAPK (64). The Wnt signaling pathway has remained elusive to therapeutic targeting, due to issues with protein-binding sites (β-Catenin) or significant side effects (65, 66). Beyond consideration for signaling pathways, sex differences have been noted in treatment response. Women have better outcomes than men, by virtue of slow-growing HCC that presents early enough for curative therapy (67, 68). Our studies could inform a potentially synergistic targeting of Wnt signaling, under sex hormone receptor modulation, alongside the current first-line therapy targeting other less dysregulated pathways in HCC.

The current study also represents a large integration of publicly available gene expression data in HCC, covering 36 studies and 1,268 patient sample profiles. We integrated gene expression data from PubMed and the Gene Expression Omnibus enabled the identification of the most consistently deregulated genes involved in hepatocarcinogenesis as detected by transcriptomics. These systematically curated data are made available in our Cancer Data Integration Portal (http://ophid.utoronto.ca/CDIPLiver) and will serve as a public resource for analyzing integrated HCC high-throughput gene expression data.

One key study limitation was that most HCC samples in this integrative analysis came from patients who had undergone hepatectomy, which we discovered was the nature of most publicly available gene expression data. The molecular features of different HCC stages could potentially differ, and ESR1 may not be central in those contexts.

Additionally, estrogen receptor signaling likely has an impact on other aspects of HCC biology, but we chose to focus on Wnt/B-catenin given the impact of ER-a agonist treatment on this critical HCC pathway.

In conclusion, we provide a mechanism by which ESR1 plays an important role in the sexual dimorphism of HCC, by inhibiting Wnt/β-catenin signaling, the most commonly dysregulated pathway in HCC. Our results suggest that ER-α colocalizes with the transcription factor β-catenin to inhibit transcription of pro-oncogenic genes. Our study findings serve to enhance our understanding of the mechanistic basis for protection against HCC incidence and progression in women as compared to men.

## Data Availability Statement

The datasets presented in this study can be found in online repositories. The names of the repository/repositories and accession number(s) can be found in the article/**Supplementary Material**.

## Ethics Statement

The animal study was reviewed and approved by University of Toronto AUP 20012289, University Health Network AUP 6105.

## Author Contributions

Conceptualization: MB and IJ. Methodology: MB, EP, CP, CB, MK, OA, and IJ. Investigation: MB, EP, CP, MAn, MAb, MK, OA, and IJ. Visualization: MB and IJ. Funding acquisition: MB and IJ. Project administration: MB, EP, and IJ. Supervision: MB and IJ. Writing – original draft: MB, EP, CP, and IJ. Writing – review and editing: MB, EP, CP, MAn, CB, MK, and IJ.

## Funding

Canadian Institutes for Health Research Fellowship (MB),Toronto General and Western Hospital Research Foundation (MB), Canadian Liver Foundation (MB), Natural Sciences Research Council (IJ), Canada Foundation for Innovation (IJ), Ontario Research Fund (IJ), IBM and Ian Lawson van Toch Fund (IJ).

## Conflict of Interest

The authors declare that the research was conducted in the absence of any commercial or financial relationships that could be construed as a potential conflict of interest.

## Publisher’s Note

All claims expressed in this article are solely those of the authors and do not necessarily represent those of their affiliated organizations, or those of the publisher, the editors and the reviewers. Any product that may be evaluated in this article, or claim that may be made by its manufacturer, is not guaranteed or endorsed by the publisher.
