# Supplementary material for: Estrogen Receptor 1 Inhibition of Wnt/β-Catenin Signaling Contributes to Sex Differences in Hepatocarcinogenesis
Source: Front Oncol. 2021 Nov 22;11:777834. doi: 10.3389/fonc.2021.777834 (PMC8645636; doi:10.3389/fonc.2021.777834)
Supplement: Supplementary file 1 [file DataSheet_1.docx]

Supplementary Material

*Data collection, analysis, and database compiling*

The following MeSH Terms were used for the search in PubMed: http://www.ncbi.nlm.nih.gov/PubMed) using the following MeSH terms: “("carcinoma, hepatocellular"[MeSH Terms] OR ("carcinoma"[All Fields] AND "hepatocellular"[All Fields]) OR "hepatocellular carcinoma"[All Fields] OR ("hepatocellular"[All Fields] AND "carcinoma"[All Fields])) AND ("gene expression"[MeSH Terms] OR ("gene"[All Fields] The following MeSH Terms were used for the search in PubMed: http://www.ncbi.nlm.nih.gov/PubMed) using the following MeSH terms: “("carcinoma, hepatocellular"[MeSH Terms] OR ("carcinoma"[All Fields] AND "hepatocellular"[All Fields]) OR "hepatocellular carcinoma"[All Fields] OR ("hepatocellular"[All Fields] AND "carcinoma"[All expression"[MeSH Terms] OR ("gene"[All Fields] AND "expression"[All Fields]) OR "gene expression"[All Fields]) AND ("humans"[MeSH Terms] OR "humans"[All Fields]) AND English[All Fields] NOT ("review"[Publication Type] OR "review literature as topic"[MeSH Terms] OR "reviews"[All Fields]) AND ("2002/01/01"[PDAT]: "2016/12/31"[PDAT]).

The following MeSH Terms were used for the search in GEO: ("carcinoma, hepatocellular"[MeSH Terms] OR hepatocellular carcinoma[All Fields]) AND "Homo sapiens"[porgn] AND "Expression profiling by array"[Filter].

***Cell lines***

HepG2 cells (ATCC® HB-8065), known to have exon 3 mutated of the β-catenin gene (CTNNB1) leading for a costitutively active state of CTNNB1^1^ were maintained in DMEM (supplemented with 10% fetal bovine serum) with 100 units/ml penicillin plus 100 μg/ml streptomycin (Invitrogen) at 37 °C in a 5% CO2 atmosphere. Before any experiment involving the exposure to estrogen, cells were kept for at least 72 hours in a phenol red-free DMEM (Thermo Fisher) media with 10% FBS that was charcoal-stripped (Thermo Fisher).

HeLa and MCF7, used as internal control for the immunofluorescence experiments were maintained in DMEM and EMEM, respectively, supplemented with 10% fetal bovine serum and 100 units/ml penicillin plus 100 μg/ml streptomycin (Invitrogen) at 37 °C in a 5% CO2 atmosphere.

***Cell transfection***

Transfections were carried out using liposomes (Lipofectamine 3000, Life Technologies) (Carlsbad CA, USA) followed by treatment with Estradiol (E2, at 10^-8^M) Sigma Aldrich for 18–72 hours. Cells were seeded in a 96-well plate, for proliferation assay with IncuCyte Zoom ® system (Essen Bioscience, Ann Arbor, MI, USA) and viability assessment with the Alamar Blue assay. Cells were separately seeded in a 6-well plate for gene expression profiling. For immunofluorescence, cells were seeded in a 4-well- chamber slide system (Nunc® Lab-Tek® Chamber Slide™). HepG2 cells were at 70% confluence at the time of transfection as per manufacturer’s instructions and transfected with pCMV6-AC-GFP-ESR1 (Origene, Rockville, MD, USA) or the empty control vector pCMV6-AC-GFP Mammalian Expression Vector (Origene, Rockville, MD, USA). Plasmid DNA-lipid complexes were prepared as suggested by the manufacturer: 100 ng of DNA for 96-well plate transfection protocol, 2.5 µg of DNA for the 6-well plate transfection and 1 µg of DNA for the 4-chamber slide transfection. The concentrations of E2 (10^-8^ M) used in these experiments have been described previously^2, 3^. Exposure to E2 or vehicle was performed 4 hours from the transfection for all transfection or non-transfection conditions.

HeLa and MCF7, used as internal control for the immunofluorescence experiments were maintained in DMEM and EMEM, respectively, supplemented with 10% fetal bovine serum at 37°C in a 5% CO2 atmosphere.

***Real-Time PCR***

RNA was isolated from cell lines or liver samples using the Qiagen RNeasy mini kit (Qiagen, Valencia, CA) according to manufacturer’s instructions. cDNA was synthesized using the QuantiTect Reverse Transcription Kit (Qiagen, Valencia, CA). RT-PCR was performed on the Step One Plus platform (Applied Biosystem, Carlsbad, CA) using the SYBR Green Real-Time PCR Master Mix (Thermo Fisher, Waltham, MA) with the following oligonucleotide primers for human:

*ESR1*-F: 5’-GCCAACGCGCAGGTCTA-3’

*ESR1*-R: 5’-GCCGCAGCCTGAGA-3’

*CCND1*-F: 5'-CATCTACAGCGACAACTCCATC-3’

*CCND1*-R: 5'-TCTGGCATTTTGCAGAGGAAG-3’

*MYC-*F: 5’-AAACAGAAACTTGAACAGCTAC-3’

*MYC-*R: 5’-ATTTGAGGCAGTTTACATTATGG-3’

*HPRT-*F: 5’-TCA GGC AGT ATA ATC CAA AGA TGG T-3’

*HPRT*-R: 5’-CTT CGT GGG GTC CTT TTC AC-3’

All data were normalized to the average C_T_ value for *HPRT*. The ΔΔ Ct method was used to calculate the expression fold-change of the target gene in the agonist mice compared to the control group.

***IncuCyte Zoom Proliferation assessment***

HepG2 cells seeded onto a 96-well plate (at a density of 4 x10^3^/well) were transfected and kept up to 90 hours at standard culture conditions in the IncuCyte ZOOM® system, which enables live-cell imaging, analysis and automated quantification of cell behavior over time. The platform was set to automatically acquire images every 4 hours. Kinetic measures of cell growth were based on area confluence metrics (obtained by real images from the plate) and were obtained using IncuCyte S3 Software.

***Alamar Blue Viability assay***

The viability of HepG2 transfected with ESR1 or control vector exposed to Estradiol (E2 at 10^-8^M) or vehicle was measured at 72 hours after transfection and compared to control conditions. Alamar Blue Cell Viability Reagent (Thermo Fisher, Waltham, MA, U.S.A.) was added to the cells and incubated for 4 hours to convert resazurin to resorufin. Fluorescence signal, as well as changes in color and fluorescence due to variations in cell viability, were measured by Varioskan™ LUX multimode microplate reader (wavelength 544/590) from Thermo Fisher. Three independent experiments were performed.

***Immunofluorescent microscopy for ESR1 and β-catenin cellular localization***

Cellular localization of ESR1 and β-catenin in HepG2 cells transfected with ESR1 or control vector and exposed to Estradiol (E2 at 10^-8^M) or vehicle was measured at 72 hours after transfection and compared to control conditions (non-transfected HepG2 cells exposed to E2 or vehicle). Cells were rinsed with PBS and fixed with 4% paraformaldehyde for 10 min at room temperature followed by permeabilization with 0.1% Sodium Citrate plus 0.1% Triton X-100. For dual staining, the cells were incubated with anti-ESR1 (1:200) antibody (Abcam, ab32063, rabbit monoclonal) and anti-β-catenin (1:100) antibody (Thermo Fisher, 13-8400, mouse monoclonal) for 2 h at room temperature. Cells were then washed with cold PBS three times for 3 min each and incubated with FITC-labeled anti-rabbit secondary antibody (1:800) (Invitrogen) and Cy3 -labeled anti-mouse secondary antibody (1:800) was incubated for 1 h at room temperature. Nuclei were stained with SlowFade Gold Antifade Mountant with DAPI (Thermo Fisher S36938). Cells were examined by fluorescence microscopy (Olympus America Inc, Center Valley, PA) and images from six randomly selected microscopic fields of cells were acquired.

***Luciferase Assay***

Cells were co-transfected using Lipofectamine 3000 (Life Technologies), as previously reported^4^, with 100 ng M50 Super 8×TOPFlash (Addgene), and 10 ng pRL-null Renilla luciferase Promega) in 96 wells to measure β-catenin transcriptional activity with or without co-transfection of ESR1 vector (100 ng, Origene). Cells were then exposed to Estradiol (E2 at 10^-8^M). The Dual-Luciferase Reporter Assay System (Promega) was used at 72 hours to determine firefly and renilla luciferase activity according to the manufacturer's instructions and as previously reported^4^.

***HepG2 Gene Expression Array***

RNA was obtained from N=3 independent experiments of transfected HepG2 or control conditions using RNeasy mini kit from Qiagen.

Integrity of DNA and RNA was assessed by Agilent 2100 Bioanalyzer (Agilent, Santa Clara, CA, USA). 500 nanograms of RNA were used for analysis with Affymetrix Human 2.0 ST Array platform. Raw array data were processed using the rma function in the Affy package^5^ in R version 3.6.

***β-catenin*** ***target gene identification***

In order to identify the possible target genes transcribed by *β-catenin* ChEA (downloaded April 2018)^6^ and Cistrome (downloaded May 2018)^7^ databases were used.

***Immunohistochemical Validation***

Six HCC mouse samples (N=2 per group, agonist and control both male and female) were included for IHC validation using anti-β-catenin antibody (Abcam, ab224803, rabbit monoclonal), anti-cyclin-D1 antibody (Abcam, ab16663, rabbit monoclonal), anti-Ki67 antibody (Thermo Fisher RM-9106-S1 rabbit monoclonal antibody), and anti- ~~ER-a~~ ER-αantibody (Abcam, ab32063, rabbit monoclonal). Formalin-fixed Paraffin-embedded (FFPE) sections (5 um) were pre-treated for antigen retrieval following the manufacturer’s instructions. The dilution for anti-β-catenin, anti-cyclin-D1 and anti-Ki-67 antibody was 1:400, for anti- ~~ER-a~~ ER-α antibody was 1:100 and an anti-rabbit was used as the secondary antibody. The complex was then visualized with hydrogen peroxide substrate and 3, 3’-diaminobenzidine tetrahydrochloride (DAB) chromogen. Slides were then counterstained with Harris Hematoxylin^®^ scanner at 20x magnification and viewed with the Aperio ImageScope^®^ v12.4.3.5008.

Supplementary Figures

S

Supplementary Figure 1: Flow chart illustrating the paper selection process and exclusion criteria.

**Supplementary Figure 2:** Exponential rise in the number of interactions after degree 250, which was chosen as the threshold for significant number of interactions.


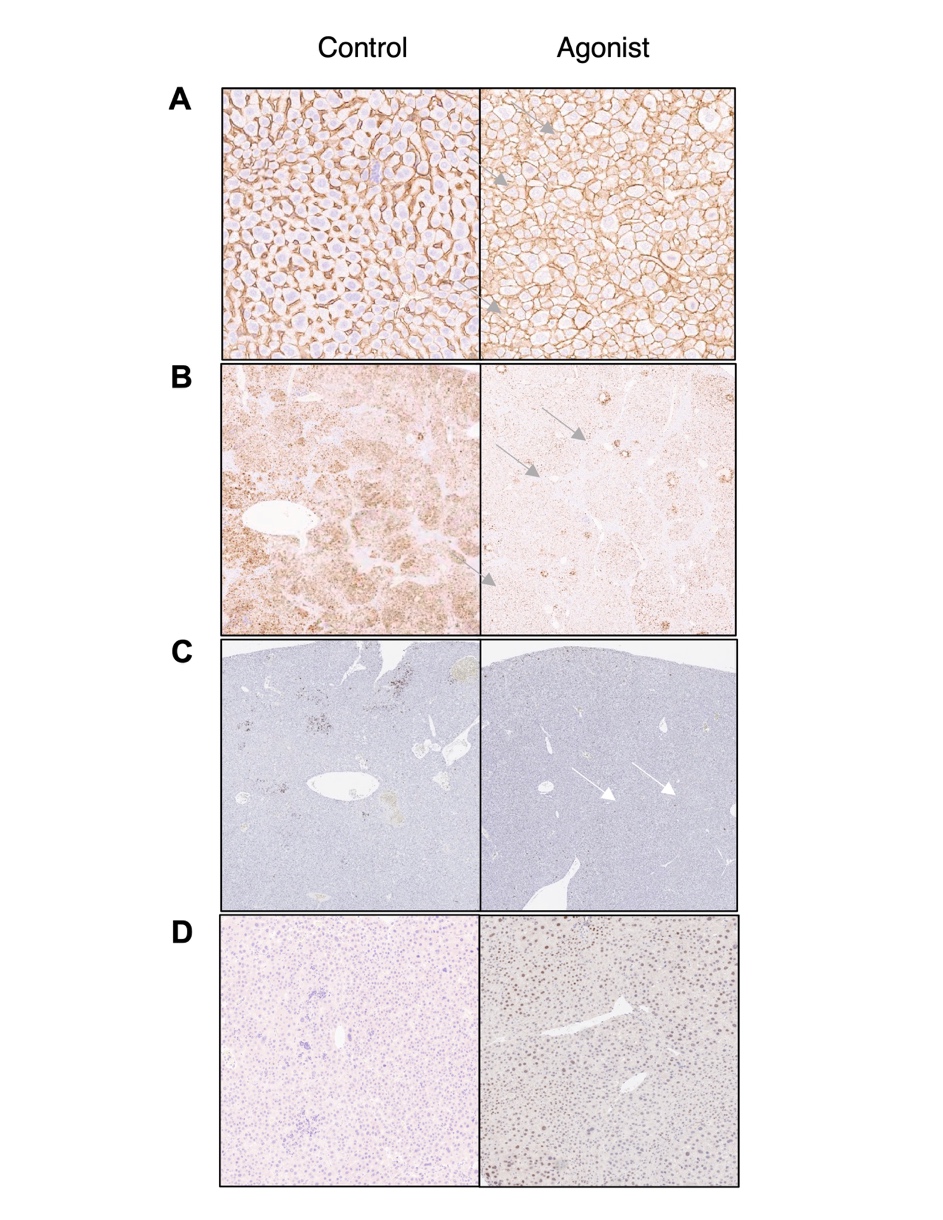


**Supplementary Figure 3:** ER-α **agonist decreases β-catenin, cyclin-D1 and Ki67 in HCC (A)** Immunohistochemistry for β-catenin in liver sections from the control with 90% tumor burden and agonist mice with 70% tumor burden (Magnification 40X). The images show a different intensity of staining between the groups. (**B)** Immunohistochemistry for cyclin D1 in liver sections from the control with 90% tumor burden and agonist mice with 70% tumor burden (Magnification 2X). The images show strong differences in tissue distribution and intensity of staining between the groups. (**C)** Immunohistochemistry for Ki67 in liver sections from the control with 90% tumor burden and agonist mice with 60% tumor burden (Magnification 2X). **(D)** Immunohistochemistry for ER-α in liver sections from the control with 90% tumor burden and agonist mice with 70% tumor burden (Magnification 2X). There is an overall difference in ER-αdistribution between the two groups, with more intense nuclear staining in the agonist group compared to the control.

**
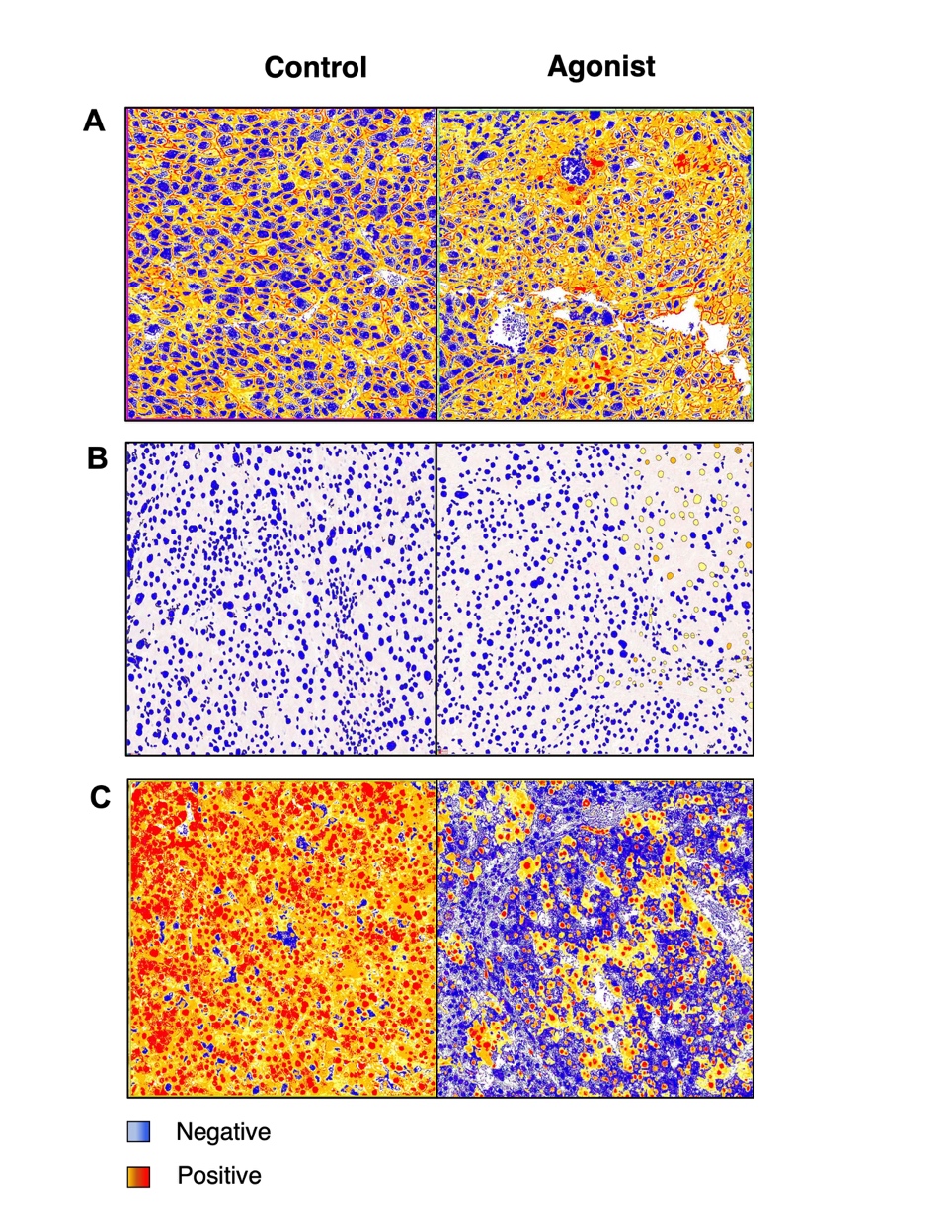
**

**Supplementary Figure 4:** Agonist female mice show an increased percent of positive nuclei for ER-α and β-catenin with a decreased nuclear positivity for cyclin-D1. **(A)** Nuclei positivity quantification performed on Immunohistochemistry section for β-catenin in liver sections from the control with 90% tumor burden and agonist mice with 70% tumor burden. The images show a different positivity of staining between the groups. **(B)** Nuclei positivity quantification performed on immunohistochemistry for ER-α in liver sections from the control with 90% tumor burden and agonist mice with 70% tumor burden. The images show a different positivity of staining between the groups. **(C)** Nuclei positivity quantification performed on immunohistochemistry for cyclin-D1 in liver sections from the control with 90% tumor burden and agonist mice with 70% tumor burden. The images show a different positivity of staining between the groups.

**Supplementary Figure 5: ESR1 overexpression decreases β-catenin target gene expression in HepG2 cells**

**(A)**Venn Diagram shows 66 proteins members of Wnt signaling to be protein interactors of ESR1, including β-catenin (CNNTB1) and LEF1. **(B)** ESR1 mRNA expression level increased in HepG2 cells overexpressing ESR1 and exposed to E2 compared to the control group. Mean with SD values from n=3 independent experiments are represented in the graph. ****p<0.0001, Student’s t-test. **(C)** CCND1 mRNA expression level decreased in HepG2 cells overexpressing ESR1 and exposed to E2 compared to the control group. Mean with SD values from n=3 independent experiments are represented in the graph. **p=0.0019, Student’s t-test. **(D)** MYC mRNA expression level decreased in HepG2 cells overexpressing ESR1 and exposed to E2 compared to the control group. Mean with SD values from n=3 independent experiments are represented in the graph. **p=0.0023, Student’s t-test

**Supplementary Figure 6: Survival curves based on expression of ESR1 (RNAseq ID 2099) in the TCGA dataset**. **(A)** Recurrence-Free Survival according to ESR1 status on 316 patients. **(B)** Disease Free Survival according to ESR1 status on 362 patients. **(C)** Progression-Free Survival according to ESR1 status on 370 patients. Separation of patients by ESR1 expression in Kaplan-Meier plotter was based on the best cut off computed from all the possible cut off values between the lower and upper quartile.

**Supplementary Tables**

**Supplementary Table 1a:** List of the 19 papers identified with HCC Gene Expression Data available on GEO.

**Supplementary Table 1b:** List of the 17 papers identified with HCC Gene Signatures.

Supplementary Table 1c: Clinical characteristics retrieved from the 19 papers including HCC gene expression datasets.

**Supplementary Table 2:** List of the dysregulated genes identified in the 19 papers with HCC Gene Expression Data retrieved from GEO.

**Supplementary Table 3**: Betweenness calculation for the proteins presenting more than 250 interactions.

**Supplementary Table 4**: List of dysregulated genes identified in ESR1 overexpressing HepG2 cells.

**Supplementary Table 5:** Pathway enrichment analysis performed on the dysregulated genes identified in ESR1 overexpression in HepG2 cells.

**Supplementary Table 6:** List of the dysregulated genes identified in the agonist mice compared to the control mice.

**Supplementary Table 7:** Pathway enrichment analysis performed on the up- or down-regulated genes identified in the agonist mice compared to the control mice.

**Supplementary Table 8:** List of the target genes predicted by ChEA and Cistrome to be transcribed by β-catenin (CNNTB1).

**Supplementary Table:** List of the 19 papers identified with HCC Gene Expression Data available on GEO.

**Supplementary Table 1b:** List of the 17 papers identified with HCC Gene Signatures.

**Supplementary Table 1c:** Clinical characteristics retrieved from the 19 papers including HCC gene expression datasets.

**Supplementary Table 2-8**

Lists are too Large to display in this format.

References:

1. Lachenmayer A, Alsinet C, Savic R, et al. Wnt-pathway activation in two molecular classes of hepatocellular carcinoma and experimental modulation by sorafenib. *Clin Cancer Res*. Sep 15 2012;18(18):4997-5007. doi:10.1158/1078-0432.CCR-11-2322

2. Koutsodontis G, Kardassis D. Inhibition of p53-mediated transcriptional responses by mithramycin A. *Oncogene*. Dec 9 2004;23(57):9190-200. doi:10.1038/sj.onc.1208141

3. Lai MD, Jiang MJ, Wing LY. Estrogen stimulates expression of p21Waf1/Cip1 in mouse uterine luminal epithelium. *Endocrine*. Apr 2002;17(3):233-9. doi:10.1385/ENDO:17:3:233

4. Fako V, Yu Z, Henrich CJ, Ransom T, Budhu AS, XW. W. Inhibition of wnt/β-catenin Signaling in Hepatocellular Carcinoma by an Antipsychotic Drug Pimozide. *Int J Biol Sci 2016 Apr 28*. 12(7):768-75.

5. Gautier L, Cope L, Bolstad BM, Irizarry RA. affy--analysis of Affymetrix GeneChip data at the probe level. *Bioinformatics*. Feb 12 2004;20(3):307-15. doi:10.1093/bioinformatics/btg405

6. Lachmann A, Xu H, Krishnan J, Berger SI, Mazloom AR, A. Ma. ChEA: transcription factor regulation inferred from integrating genome-wide ChIP-X experiments. *Bioinformatics*. 2010;Oct 1;26(19):438-44.

7. Liu T, Ortiz JA, Taing L, et al. Cistrome: an integrative platform for transcriptional regulation studies. Genome Biol. 2011 Aug 22;12(8):R8. *Genome Biol* 2011;Aug 22;12(8):R83.
